# Supplementary material for: Assessment of the fatality rate and transmissibility taking account of undetected cases during an unprecedented COVID-19 surge in Taiwan
Source: BMC Infect Dis. 2022 Mar 20;22:271. doi: 10.1186/s12879-022-07190-z (PMC8934571; doi:10.1186/s12879-022-07190-z)
Supplement: Supplementary file 1 — Additional file 1. Figure S5. [file 12879_2022_7190_MOESM1_ESM.docx]

**Additional file 1**

**Assessment of the fatality rate and transmissibility taking account of undetected cases during an unprecedented COVID-19 surge in Taiwan**

**Hsiang-Yu Yuan^1,2,^*, M. Pear Hossain^1^, Tzai-Hung Wen^3^, Ming-Jiuh Wang^4^**

^1^ Department of Biomedical Sciences, Jockey Club College of Veterinary Medicine and Life Sciences, City University of Hong Kong, Hong Kong SAR, China

^2^ Centre for Applied One Health Research and Policy Advice, City University of Hong Kong,

Hong Kong SAR, China

^3^ Department of Geography, National Taiwan University, Taipei City, Taiwan

^[4](https://www.researchgate.net/institution/National_Taiwan_University_Hospital)^ [Department of Anesthesiology, National Taiwan University Hospital, National Taiwan University, Taiwan](https://www.researchgate.net/institution/National_Taiwan_University_Hospital)

*Correspondence to: Hsiang-Yu Yuan sean.yuan@cityu.edu.hk

Hsiang-Yu Yuan and M. Pear Hossain contributed equally to this article.

# Supplementary Materials

## Establishing the relationship between mobility and effective reproduction number

Daily mobility data (Figure S3A) were obtained from Google mobility report^26^ and were normalized after setting the mobility index on May 11 (first day of the start of the outbreak) as 1 and the value -100 as 0. The normalized mobility index ranged between 0 and 1, where higher values represent greater mobility. To compare and validate the estimated $R_{t}$, we used a generalized linear model for Gaussian distribution with identity link function. Mobility index was adjusted in the model using the following formula adopted from a recent study^8^:

$$\log\left( R_{t} \right) =\log(R_{0})+\beta\left( 1-M_{b}\left( t \right) \right). ( SEQ Equation \backslash* ARABIC 8)$$

where $R_{0}$ is the initial reproduction number obtained from $R_{t}$ at the start of the outbreak (May 11, 2021), which gave the maximum number of $R_{t}$; $M_{b}(t)$ represents the daily normalized mobility index; and $\beta$ is the regression coefficient.

## Supplementary figures


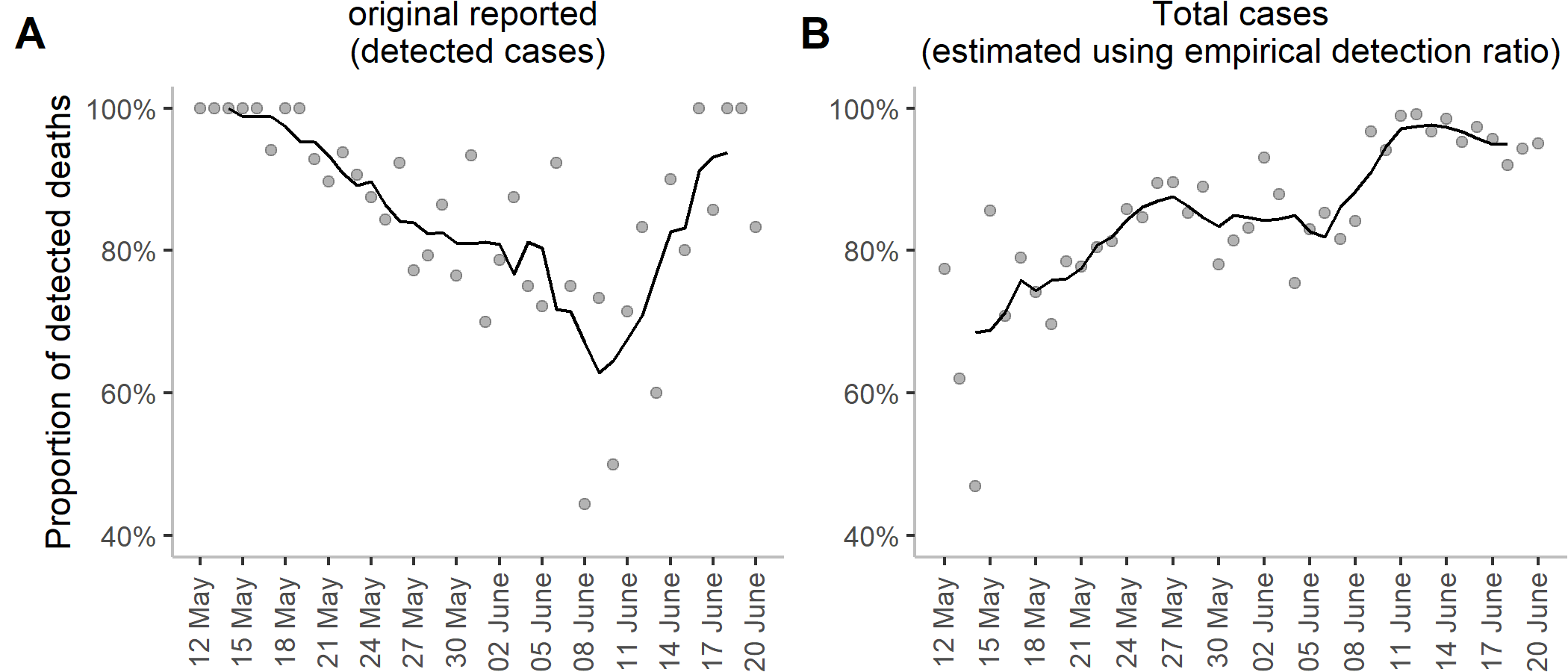


Figure S1. **(A)** Proportion of detected deaths among total reported deaths. **(B)** Proportion of detected deaths among total deaths estimated using the empirical detection ratio. In each plot, dots represent daily numbers that are observed or estimated. Solid lines represent moving average using a 5-day sliding window, centered at day 3.


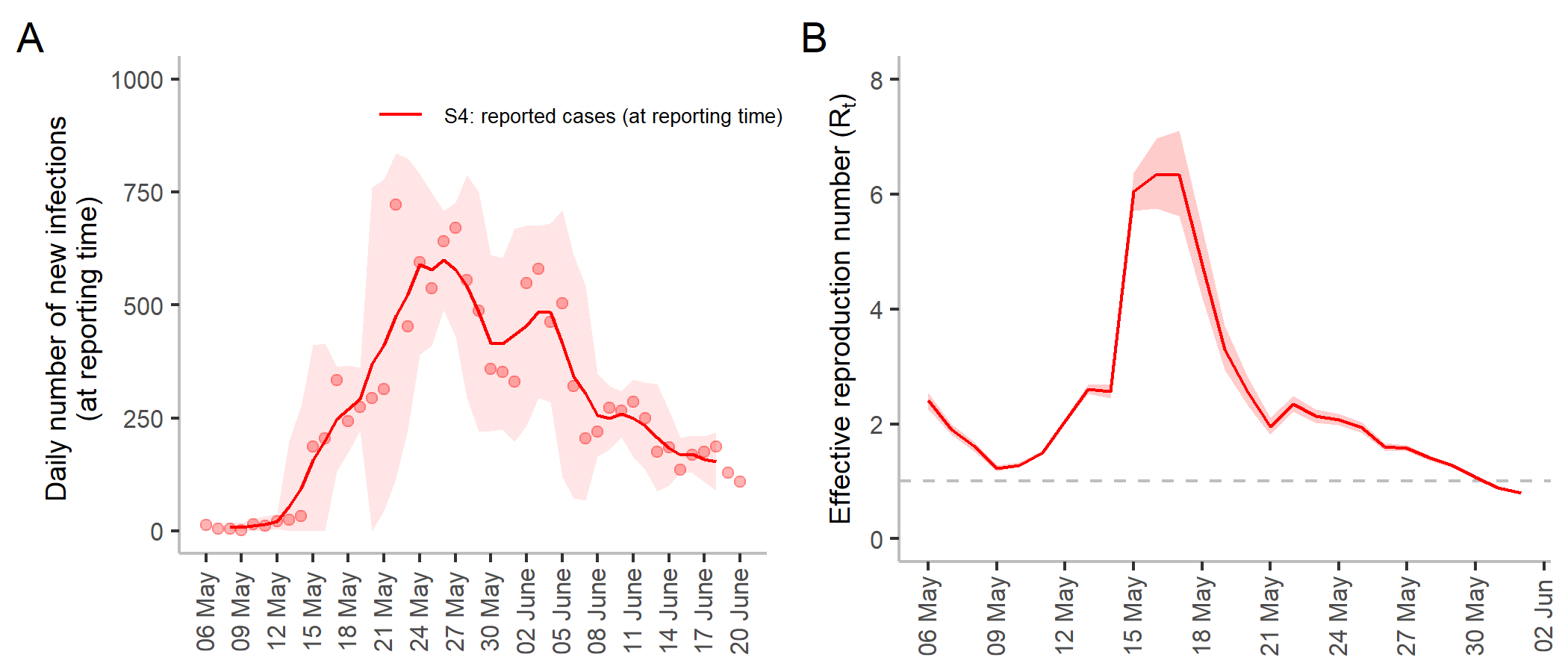


Figure S2. **(A)** Daily number of new infections at their reporting time. Daily values are indicated by red dots (referred to as S4 in Methods). The line represents moving averages using a 5-day sliding window, centered at day 3. **(B)** Effective reproduction number estimated from (A). The solid red line represents estimated values. The shaded area represents 95% confidence intervals. The dashed line depicts the cutoff value when $R_{t}=1$. The value $R_{t}$ during the entire period (between May 6 and June 20) is given in the Supplementary Figure S4D.


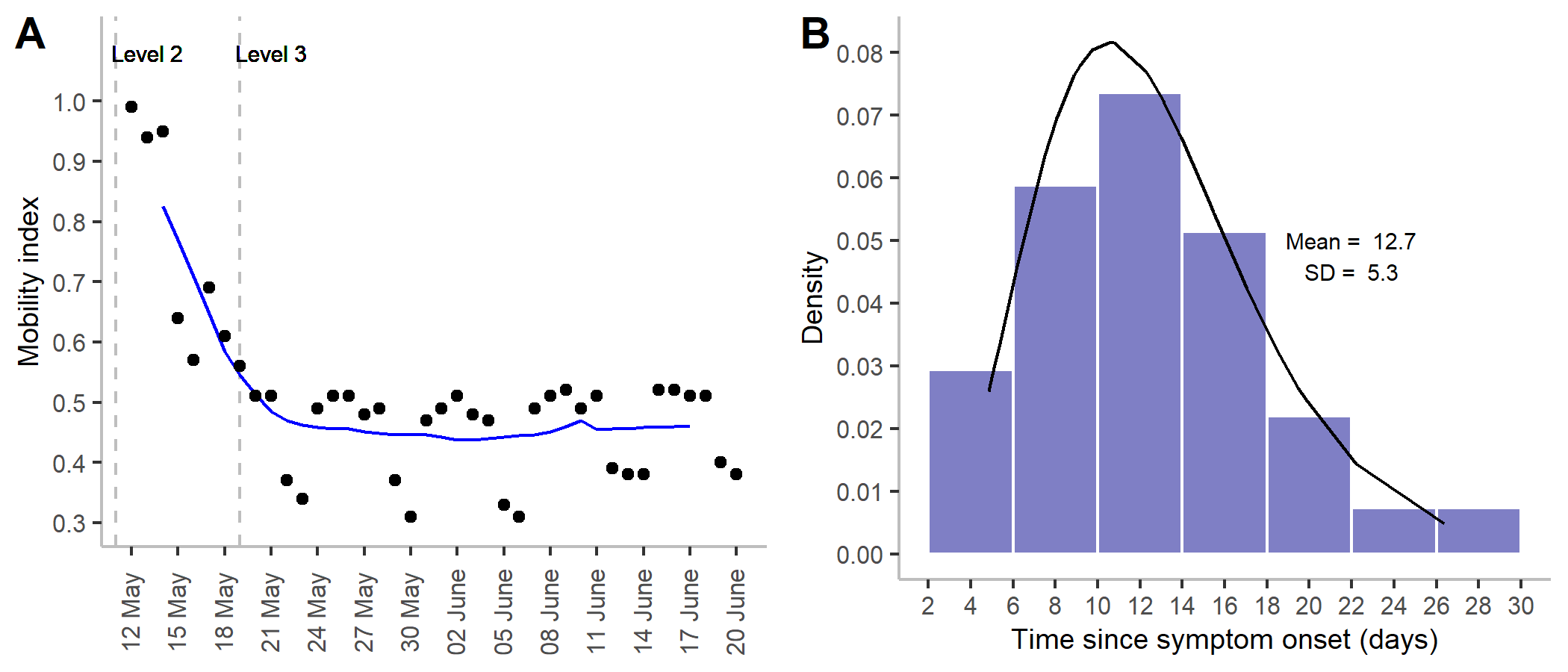


Figure S3. **(A)** Mobility index during the outbreak. The smooth line shows a 7-day moving average, whereas the dots represent the observed mobility index. The vertical dashed lines represent the implementation of level 2 and level 3 restrictions in May and June. Level 2 restrictions were started on May 11 and lasted until June 8, whereas level 3 restrictions were imposed for the duration between May 19 and May 28. **(B)** Distribution of death delay. The bars represent the observed frequency of delay distribution and line represents the fitted line for gamma distribution with mean and standard deviation 12.7 and 5.3 days, respectively.


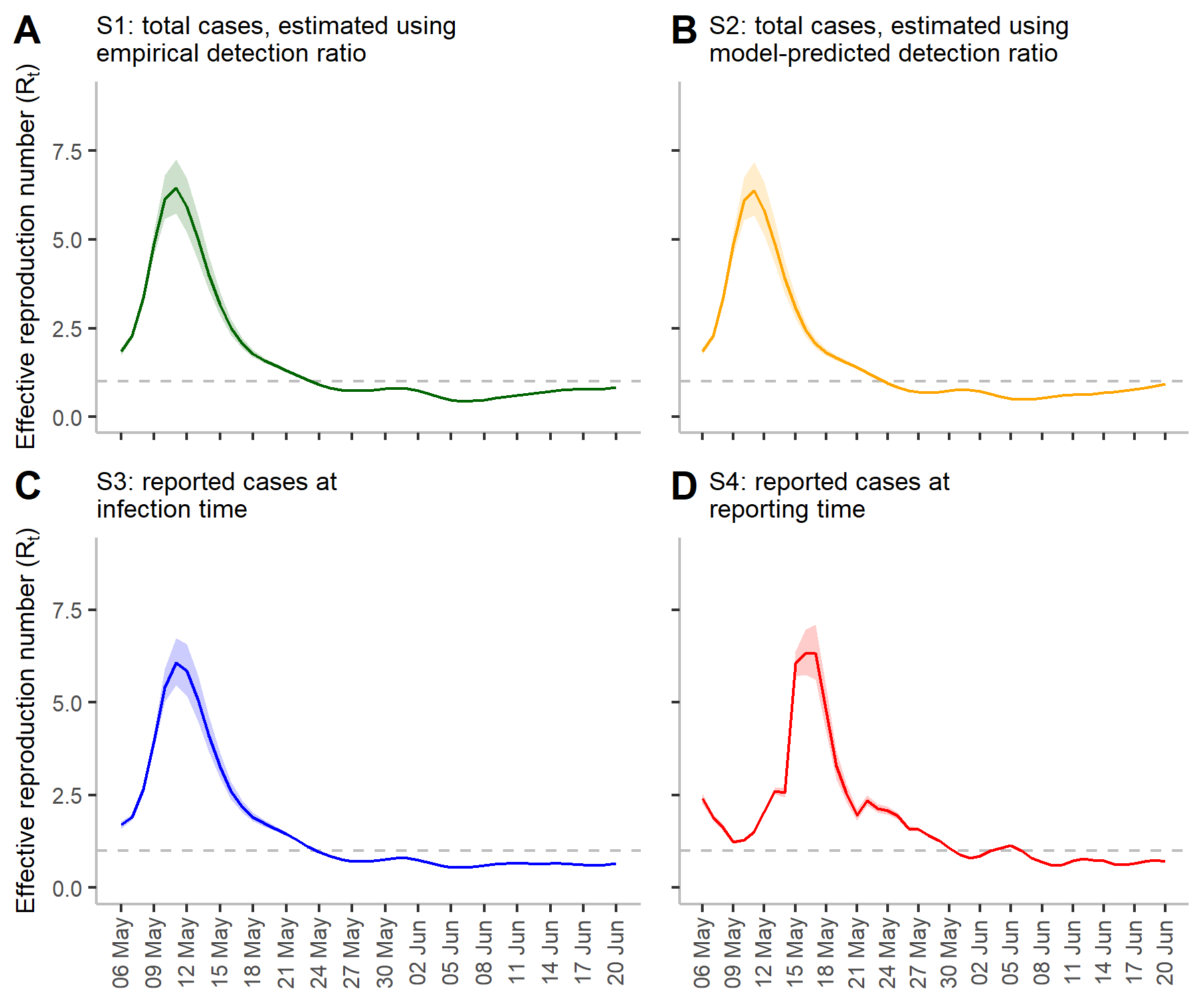


Figure S4. Effective reproduction number $R_{t}$ during the entire period between May 6 and June 20. S1 and S2 refer to the numbers of total cases at infection time. S3 and S4 refer to the numbers of reported cases at infection and reporting time, respectively. Smooth solid lines represent the estimated mean $R_{t}$, and shaded regions show the 95% confidence intervals. The dashed line depicts the cutoff value when $R_{t}=1$.


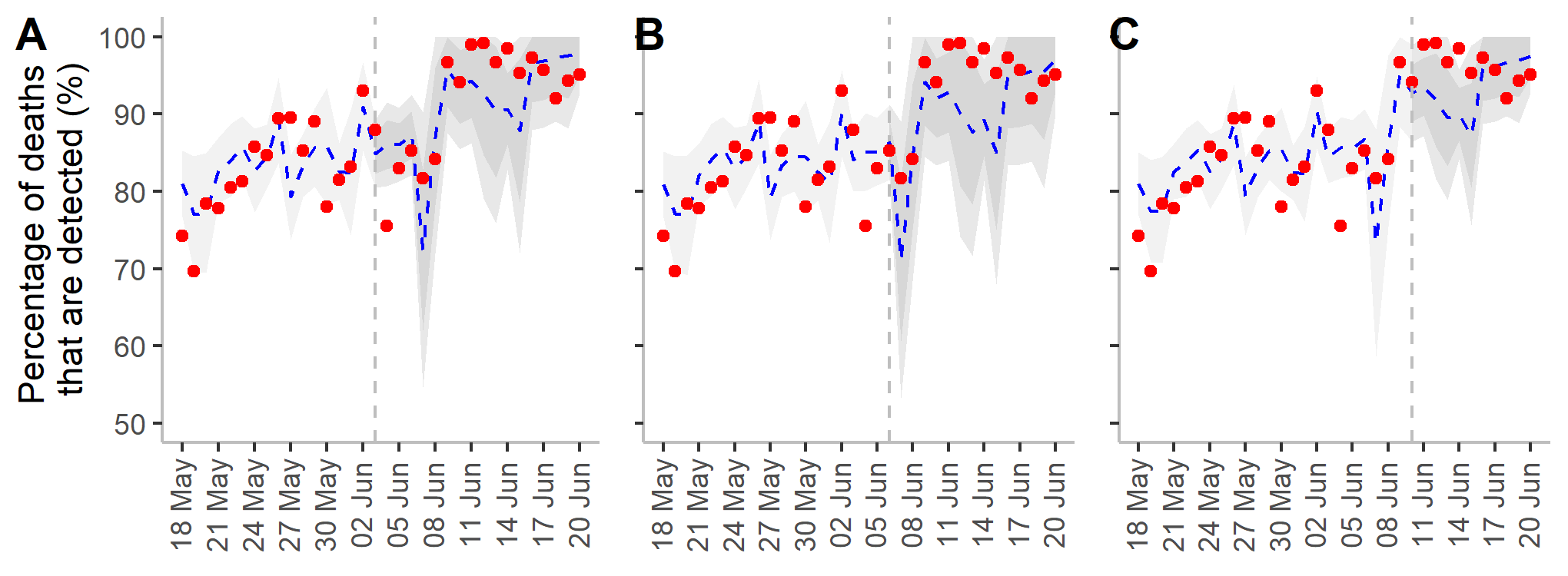


Figure S5. Model validation results. The best-fitting model was validated using different past training sets, such as the first 50% (A), 60% (B) and 70% (C) of the full data sets. Data on right hand side of the vertical line in each plot represent validation sets, 50, 40 and 30% respectively. Red circles represent the adjusted reported data. The blue dashed line represents the prediction results using the best-fitting model. The gray shaded area represents forecasted values of the proportion of detection with 75 (darker area) and 95% (lighter area) confidence intervals.

## Supplementary tables

Table S1. Validation of the estimates of instantaneous reproduction number using mobility adjusted regression model between May 11 and May 24 when $R_{t}$ reached one. The moving average of mobility using a 7-day sliding window, centered at day 4, was considered as the predictor. AIC represents the Akaike information criterion. $\Delta AIC$ shows the differences between the smallest AIC and AIC of the ith model. We rechecked the values for an extended period until May 27, when $R_{t}$ reached a minimum. In this case, $R_{t}$, estimated under scenario S1, showed the best fit of the mobility data with minimum AIC -13.31 (data is not presented in this table), whereas scenario S2 was treated as the second-best with AIC -8.55. The difference between the AIC of these two scenarios was 4.75.

| **Date** | **Type of data** $\boldsymbol{R}_{\boldsymbol{t}}$ **estimated from** | **Validation window**  **11 May- 24 May** | |
| --- | --- | --- | --- |
|  |  | **AIC** | $\boldsymbol{\Delta}$**AIC** |
| At infection time | S1: Total cases estimated using the empirical detection ratio | **-27.93** | **0.00** |
|  | S2: Total cases estimated using the model-predicted detection ratio | -27.20 | 0.73 |
|  | S3: Reported cases | -21.70 | 6.23 |
| At reporting time | S4: Reported cases | 27.56 | 55.49 |
